# Open-label, controlled, phase 2 clinical trial assessing the safety, efficacy, and pharmacokinetics of INM004 in pediatric patients with Shiga toxin-producing *Escherichia coli*–associated hemolytic uremic syndrome

**DOI:** 10.1007/s00467-024-06583-3

**Published:** 2024-11-12

**Authors:** Alicia Fayad, Iliana Principi, Alejandro Balestracci, Laura Alconcher, Paula Coccia, Marta Adragna, Oscar Amoreo, María Carolina Bettendorff, María Valeria Blumetti, Pablo Bonany, María Laura Flores Tonfi, Luis Flynn, Lidia Ghezzi, Jorge Montero, Flavia Ramírez, Claudia Seminara, Ángela Suarez, Ana Paula Spizzirri, Marta Rivas, Mariana Pichel, Vanesa Zylberman, Linus Spatz, Carolina Massa, Marina Valerio, Santiago Sanguineti, Mariana Colonna, Ian Roubicek, Fernando Goldbaum, on behalf of INM004 Study Group

**Affiliations:** 1 Hospital de Niños Ricardo Gutiérrez, Ciudad Autónoma de Buenos Aires, Argentina; 2 Hospital Pediátrico Dr. Humberto J. Notti, Mendoza, Argentina; 3 Hospital General de Niños Pedro de Elizalde, Ciudad Autónoma de Buenos Aires, Argentina; 4 Hospital Interzonal Regional Dr. José Penna, Buenos Aires, Argentina; 5 Hospital Italiano de Buenos Aires, Ciudad Autónoma de Buenos Aires, Argentina; 6 Hospital de Pediatría S.A.M.I.C. Prof. Dr. Juan P. Garrahan, Ciudad Autónoma de Buenos Aires, Argentina; 7 Hospital de Alta Complejidad en Red El Cruce Dr. Néstor Carlos Kirchner, Buenos Aires, Argentina; 8 Sanatorio Allende, Córdoba, Argentina; 9 Clínica Zabala, Ciudad Autónoma de Buenos Aires, Argentina; 10 Establecimiento Asistencial Dr. Lucio Molas, Santa Rosa, Argentina; 11 Sanatorio Güemes, Ciudad de Buenos Aires, Argentina; 12 Sanatorio de Niños, Rosario, Argentina; 13 Hospital Interzonal Especializado Materno Infantil Don Victorio Tetamanti, Buenos Aires, Argentina; 14 Hospital Provincial Neuquén, Dr. Eduardo Castro Rendón, Neuquén, Argentina; 15 Hospital de Niños de La Santísima Trinidad, Córdoba, Argentina; 16 Hospital de Niños Sor María Ludovica, Buenos Aires, Argentina; 17 Inmunova SA, Ciudad Autónoma de Buenos Aires, Argentina; 18 National Scientific and Technological Research Council, CONICET, Ciudad Autónoma de Buenos Aires, Argentina

**Keywords:** Hemolytic uremic syndrome, Acute kidney injury, Clinical trial, Phase 2, Passive immunotherapy

## Abstract

**Background:**

Shiga toxin-producing *Escherichia coli*-associated hemolytic uremic syndrome (STEC-HUS) is a severe condition mainly affecting children. It is one of the leading causes of acute kidney injury in the pediatric population. There is no established therapy for this disease. INM004 is an anti-Shiga toxin composed of equine polyclonal antibodies. This study is aimed at assessing the safety, pharmacokinetics, and efficacy of INM004 in pediatric patients with STEC-HUS.

**Methods:**

Phase 2, open-label clinical trial with an historical control arm. Patients in the treatment arm received two doses of INM004. The primary endpoints were the safety profile, pharmacokinetics, and efficacy (dialysis days) of INM004. Secondary endpoints included other kidney and extrarenal outcomes. Propensity score matching was used for efficacy comparisons between arms.

**Results:**

Fifty-seven and 125 patients were enrolled in the treatment and control arm, respectively. After propensity score matching, 52 patients remained in each arm. INM004 was well-tolerated. Eight adverse events were considered possibly related, none of which were serious or severe. In the primary efficacy endpoint, patients of the treatment arm presented a non-statistically significant difference of two dialysis days. On secondary endpoints, non-statistically significant trends toward fewer patients needing dialysis and dialysis for more than 10 days, and shorter time to glomerular filtration rate normalization, were observed favoring the treatment arm.

**Conclusions:**

INM004 showed an adequate safety profile. Efficacy non-statistically significant trends suggesting a beneficial effect in the amelioration of kidney injury were observed. These results encourage the conduction of a phase 3 study of INM004 in pediatric patients with STEC-HUS.

**Graphical Abstract:**

**Figure:**
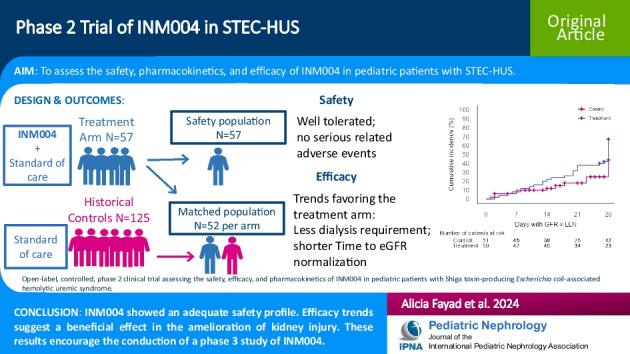
A higher resolution version of the Graphical abstract is available as Supplementary information

**Supplementary Information:**

The online version contains supplementary material available at 10.1007/s00467-024-06583-3.

## Introduction

Shiga toxin (Stx)-producing *Escherichia coli*-associated hemolytic uremic syndrome (STEC-HUS) is a form of thrombotic microangiopathy (TMA) characterized by the triad of hemolytic anemia, thrombocytopenia, and acute kidney injury (AKI) [1, 2]. It is one of the leading causes of AKI in the pediatric population and a relevant contributor to the development of chronic kidney disease (CKD) [3–6]. Approximately 60% of patients require dialysis during the acute phase of the disease and the mortality rate is around 3% [1, 3]. Children under five are the most affected group [7]. In Argentina, STEC-HUS is endemic and exhibits the highest incidence globally [8, 9].

STEC-HUS remains an orphan disease without an established treatment. Monoclonal antibodies that target Stx have shown promising preclinical and early clinical results [10–12]. INM004 is a therapy composed of F(ab´)_2_ fragments from equine polyclonal antibodies that efficiently neutralize Stx [13]. These antibodies were raised by immunizing horses with two chimeric protein particles that stabilize the B subunit of Stx1 and Stx2 in their native conformation and expose their Gb3-binding sites. Thus, the elicited antibodies would recognize many epitopes and have a strong capacity for blocking the entrance of Stx to their target cells [14]. INM004 would neutralize Stx even when the toxin circulates bound to microvesicles, a proposed key pathogenic mechanism in STEC-HUS [15].

In a phase 1 placebo-controlled trial, INM004 was well-tolerated and was not associated with serious or severe adverse events (AE) in healthy volunteers [16]. Similarly, INM004 showed good tolerance in 11 pediatric patients with Stx-positive bloody diarrhea in a phase 2/3 study for the prevention of STEC-HUS which was early terminated during the COVID-19 pandemic (NCT04132375).

The primary objective of this phase 2 study was to assess the safety, efficacy, and pharmacokinetics (PK) of INM004 in pediatric patients with STEC-HUS. The proposed mechanism of action of INM004 is the neutralization of Stx in the bloodstream to block the interaction with its receptors in the target organs. This investigation aims to provide insights into the safety and therapeutic potential of INM004, with a view to a future phase 3 trial.

## Methods

### Study design and participants

**Fig. 1:**
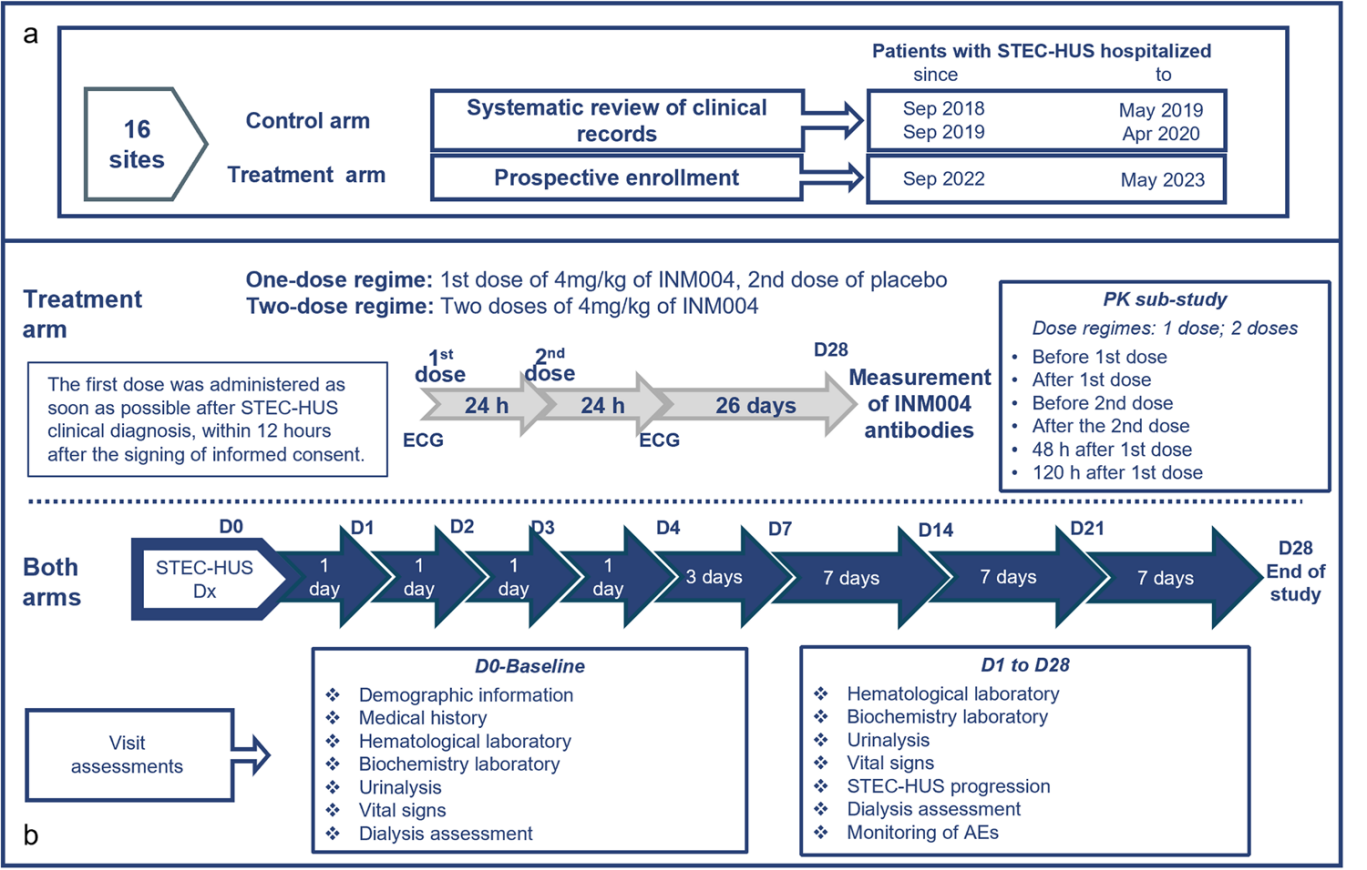
Study design and procedures. **a** Study design. **b** Visit schedule and procedures. STEC-HUS, *Escherichia coli*-associated hemolytic uremic syndrome; Dx, diagnosis; D, day; PK, pharmacokinetics; AEs, adverse events

A multicenter phase 2, open-label clinical trial of INM004 with an historical control arm was performed in 16 reference hospitals across Argentina. Eligible participants of the treatment arm were patients with STEC-HUS hospitalized at the participating centers from September 2022 to May 2023 (warm season). The control arm comprised patients corresponding to the same seasonal periods from 2018 to 2019 and 2019 to 2020, identified through a systematic review of clinical records at each participating center. The study design is detailed in Fig. 1.

Patients of the treatment arm received INM004 in addition to standard of care (SoC) treatment for STEC-HUS, and the control arm only received SoC. Overall, SoC for pediatric STEC-HUS patients was consistent between participant centers in Argentina, following specific guidelines for its management [17, 18]. Patients of the control arm were retrospectively selected by a review of all medical charts of patients hospitalized during the recruitment period for this group (Fig. 1). The evaluation of the PK of INM004 was part of a sub-study conducted in a subsample of the treatment arm (Fig. 1).

This study was prospectively registered on clinicaltrials.gov (NCT05569746). Written informed consent was provided by legal guardians of all subjects of the treatment arm. Assent of children was obtained as applicable.

### Inclusion and exclusion criteria

The same inclusion and exclusion criteria applied for both arms, except for two specific criteria related to the safety of subjects receiving an experimental treatment. In order to include patients as early as possible after STEC-HUS onset, subjects were eligible for their inclusion with two of three components of the diagnostic HUS triad: signs of kidney injury and at least one of the two hematological signs of the TMA (i.e., hemolysis and/or platelet consumption). Kidney injury was defined by serum creatinine above the upper limit of normal (ULN) for age and sex [19] or hematuria (≥ 5 red blood cells per field or ≥ 27 red blood cells/μl in the urinary sediment); hemolysis was defined by lactate dehydrogenase (LDH) above the ULN for age or presence of schistocytes in the peripheral blood smear; platelet consumption was defined by a platelet count < 150 × 10^9^/L or decrease of ≥ 50% in the platelet count within the previous 24 h. Other inclusion criteria were age between 1 and 12 years old; history of diarrhea with onset within the 13 days prior to the diagnosis of the STEC-HUS in the participating institution; a negative pregnancy test in subjects of the treatment arm who had had menarche.

Patients were excluded from the study if they met any of the following criteria. Dialysis for more than 48 h at the time of diagnosis of the STEC-HUS in the participating institution; history of chronic/recurrent hemolytic anemia, thrombocytopenia, or CKD; personal or family history of atypical HUS; suspicion of HUS secondary to infectious processes other than gastrointestinal; evidence of clinically significant chronic disease whose symptoms may have interfered with the treatment or diagnosis of STEC-HUS, at the discretion of the investigator; pregnant or breastfeeding subjects; participation in a clinical trial simultaneously or in the previous 3 months. For the treatment arm, an additional exclusion criterion was the history of anaphylaxis or previous administration of equine serum, or allergic reaction to horse exposure.

### Procedures

This study assessed two regimens of one- or two-dose of 4 mg/kg of INM004 administered as intravenous infusion during 50 min (Fig. 1). Dry weight was used for dose calculation. Therefore, weight had to be measured under normohydration conditions, or weight referred by parents could be used. While most patients were included in the two-dose regimen, the one-dose regimen was only aimed at assessing PK in a small part of the sample. Study visits were performed for all subjects on the day of diagnosis of STEC-HUS in the participating site (day 0) and on days 1, 2, 3, 4, 7, 14, 21, and 28. Hospital discharge was determined by the attending physician according to SoC, and in those cases, assessments had to continue as outpatient visits. For outpatients, visits on days 14 and 21 could be done by phone. Assessments at each visit are described in Fig. 1. A 12-lead electrocardiogram (ECG) was performed in patients of the treatment arm at baseline and 24 h after the second dose. Microbiological confirmation of STEC infection was performed by SoC procedures at each site for both arms, which could include detection of Stx by enzyme immunoassay (EIA) or detection of Stx genes by PCR in stool, fecal culture positive for *E. coli* O157, or detection of specific anti-polysaccharide IgM antibodies in serum. Additionally, IgM antibodies against *Escherichia coli* O157, O145, O121, and O103 in serum were determined in a central laboratory using commercial Chemtest® kits (CHEMLIS® E. coli Combi Glyco-iELISA) for patients of the treatment arm.

Safety and tolerability were assessed by the Data Safety Monitoring Board (DSMB) by monitoring AE and AE of special interest (AESI), laboratory values, vital signs, physical examination, and ECG. AE were coded using MedDRA version 25.1.

PK samples were collected at 6 different time points (Fig. 1). Concentrations of INM004 were determined by ELISA [16, 20]. Immunogenicity was evaluated by the presence of anti-product antibodies by ELISA in blood samples obtained at days 0 and 28 [16].

### Outcomes

Primary safety outcomes were total incidence of AE, incidence of AESI (injection site reactions and hypersensitivity reactions), and changes in laboratory parameters, vital signs, and ECG tracing after the administration of INM004 in comparison to baseline. Laboratory safety assessment included the following parameters: red blood cell count, leukocyte total count and types (lymphocytes, neutrophils, eosinophils, basophils, and monocytes), platelets, hemoglobin, hematocrit, hepatic transaminases, total and direct bilirubin, urea, creatinine, alkaline phosphatase, LDH, sodium, potassium, chloride, bicarbonate, calcium, phosphorus, pH, uric acid, albumin, and glucose. Primary PK outcomes were the serum concentrations of INM004 at different time intervals. The primary efficacy outcome was the number of dialysis days during the complete follow-up period (zero dialysis days were considered for subjects who did not dialyze).

**Fig. 2:**
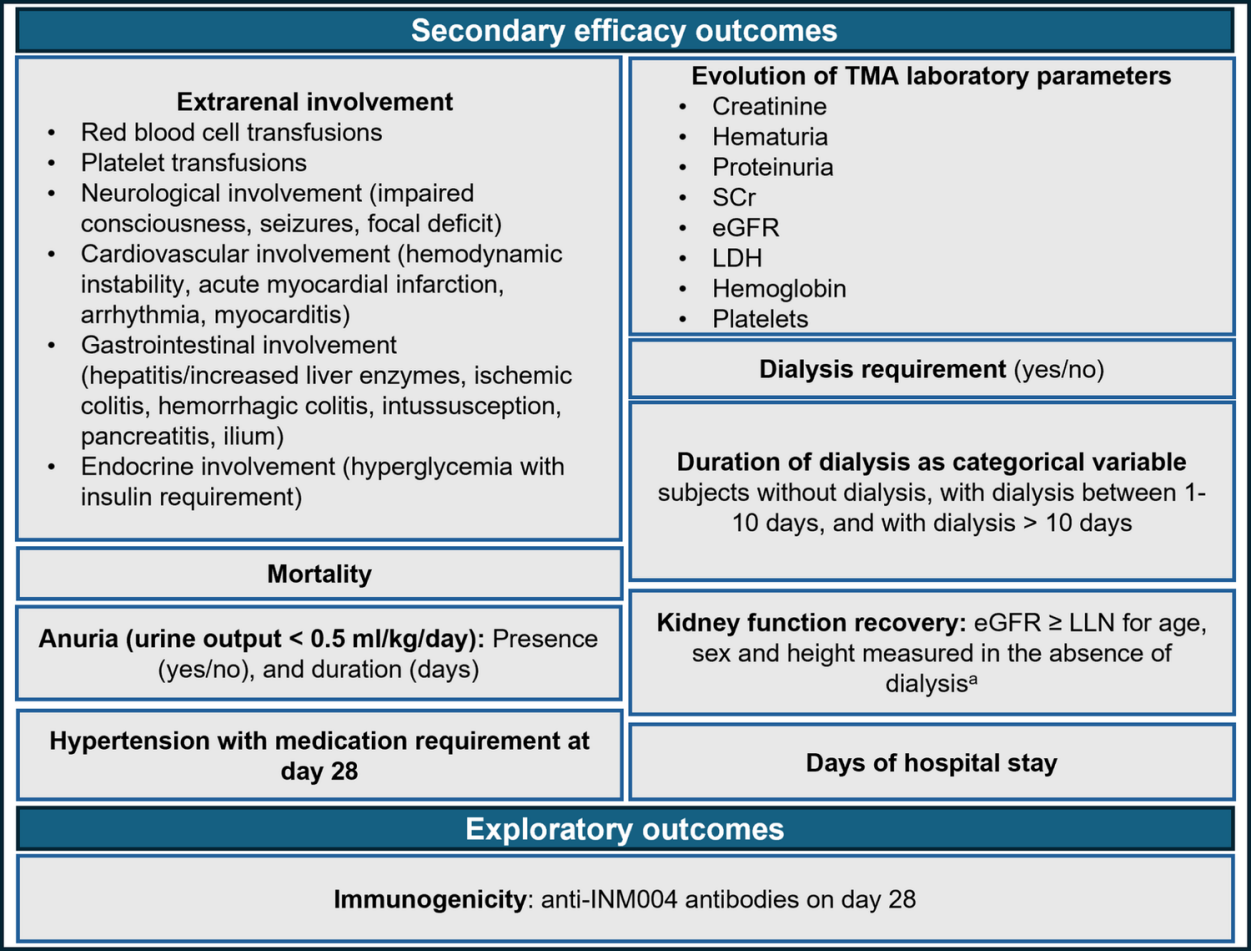
Study secondary and exploratory outcomes. TMA, thrombotic microangiopathy; eGFR, estimated glomerular filtration rate; sCr, serum creatinine; LDH, lactate dehydrogenase; LLN, lower limit of normal; a, eGFR lower limit of normal (LLN) for each subject was estimated with the Bedside Schwartz equation [36], using the serum creatinine upper limit of normal (ULN) for age and sex (eGFR_LLN_ (ml/min/1.73 m^2^) = (0.413 × height in cm)/Creatinine_ULN_ (mg/dL) for age and sex) reported by Chuang et al. (2021) [37]

Secondary and exploratory outcomes are shown in Fig. 2.

### Sample size considerations

The study aimed to evaluate the safety and PK of INM004 and provide exploratory insights into efficacy to guide the design of a phase 3 trial. One hundred patients were expected to be recruited during one warm season in the treatment arm, and 200 eligible subjects were expected for the control arm. This sample size was expected to yield a power of 78% to detect AE with an incidence of 1.5% and a power of 80% to detect a 50% difference in the primary efficacy outcome. The PK sub-study was planned to include 18 patients (12 and 6 receiving the two and one-dose regimen, respectively).

### Statistical analysis

Categorical variables were described using absolute frequencies and percentages, and quantitative variables were summarized using mean and standard deviation (SD) or median and interquartile range or range. Chi-square test or Fisher’s exact test was applied to compare categorical variables, and regression linear models were used to compare quantitative variables between the treatment and control arms. The variable dialysis days were analyzed using a negative binomial regression model to consider the overdispersion of zeros. Cox’s proportional hazard regression model was used to estimate the hazard ratio (HR) for time-to-event variables. Due to the exploratory nature of the study, no adjustments were made for multiple comparisons.

Safety analyses were descriptive. The estimation of PK parameters by non-compartmental analysis (NCA) methods was performed using Phoenix WinNonlin software (version 8.3, Certara USA, Inc., USA).

The following sets were considered for the different outcomes. Full analysis set (FAS): all subjects of the treatment arm who received at least one dose of INM004 and all subjects of the control arm; safety population (SP): all subjects from the treatment arm who received at least one dose of INM004; matched population (MP): FAS subjects selected using the propensity score matching procedure. This matching procedure (PROC PSMATCH, SAS 9.4) was used to generate a sample that matched on its baseline characteristics. The following variables at the day of diagnosis of STEC-HUS (day 0) were pre-established for the calculation of the propensity score: age, sex, days from onset of diarrhea to diagnosis of STEC-HUS, dialysis requirement, severe neurological complication (seizures, coma, or brain infarction), invasive mechanical ventilation, patient referred with > 24 h of hospitalization at the place of origin, estimated glomerular filtration rate (eGFR) [21], hemoglobin, creatinine, urea, bloody diarrhea in the prodromal period, antibiotic requirement prior to day 0, and intravascular volume expansion requirement prior to day 0. A stepwise selection of the variables was carried out by means of a logistic regression considering the arm (control and treatment) as a dependent variable and using an entry criterion of 0.8 and an output criterion of 0.3. The variables that remained in the model were finally used for calculating the propensity score. The greedy algorithm was used for the determination of the paired sample of 1:1 by the nearest similar neighbor (greedy nearest neighbor), considering a caliper equal to 0.2. Any imbalance was evaluated by the standardized mean difference (SMD). Covariates with SMD < 0.25 were considered as properly balanced.

Safety, PK, and immunogenicity evaluations were performed on the SP. All efficacy analyses were performed on the MP. As a sensitivity analysis, time-to-event analysis was repeated with multiple imputations for missing data using predictive mean matching methods. Statistical analyses were performed using SAS® software version 9.4.

## Results

### Participants

**Fig. 3:**
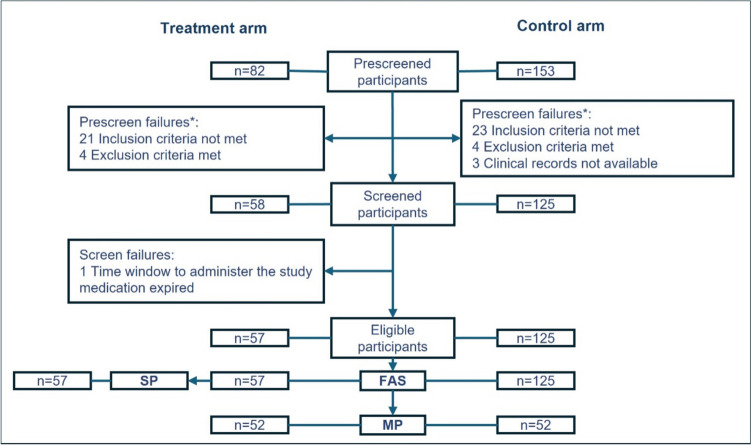
Participant disposition. FAS, full analysis set; MP, matched population; SP, safety population. *Some subjects were counted more than once because they did not meet some inclusion criteria and also met some exclusion criteria

Fifty-seven and 125 patients were included in the treatment and control arm, respectively. Fifty-two patients in each arm were part of the MP. Patient disposition is shown in Fig. 3. Ten patients were included in the PK sub-study, of which 8 received the two-dose regimen and two the one-dose regimen. Both arms of the MP were balanced in their demographic and most of their baseline clinical and laboratory parameters, except for a higher platelet count and a higher incidence of some prodromal symptoms in the treatment arm (Table 1). Demographic and baseline clinical characteristics of the SP, FAS, and PK population are presented in Supplementary Table 1, 2 and 3.

**Table 1:** Demographic and baseline characteristics of the subjects of the matched population

Demographic and baseline characteristics	Treatment arm (*n* = 52)	Control arm (*n* = 52)
Age (years), mean (SD)	2.6 (2.0)	2.9 (2.3)
Female sex, *n* (%)	26 (50)	25 (48)
Weight (kg), mean (SD)	15.2 (6.8)	15.9 (6.4)
Height (cm), mean (SD)	95.1 (15.1)	96.5 (17.1)
BMI (kg/m^2^) mean (SD)	16.2 (2.4)	16.6 (2.0)
Prodromal symptoms, *n* (%)		
Diarrhea	52 (100)	52 (100)
Bloody diarrhea	41 (79)	42 (81)
Abdominal pain	36 (69)	19 (37)
Fever	24 (46)	19 (37)
Vomiting	41 (79)	33 (58)
Therapeutic management of prodromal symptoms, *n* (%)		
Pre-admission antibiotics	10 (19)	6 (12)
Expansion	23 (44)	23 (44)
Days from diarrhea onset to diagnosis, median (Q1:Q3)	5.0 (3.0:6.0)	4.0 (4.0:6.0)
Baseline laboratory parameters, mean (SD)		
Creatinine (mg/dL)	2.4 (1.9)	2.7 (2.1)
eGFR (ml/min/1.73 m^2^)	27.6 (28.2)	26.3 (21.3)
Urea (mg/dL)	126.0 (75.0)	136.0 (82.4)
Leukocytes (10^9^/l)	18.1 (9.1)	18.5 (11.8)
Neutrophils (%)	59.3 (11.6)	58.2 (12.3)
Platelets (10^9^/l)	82.7 (57.9)	60.3 (37.5)
Hemoglobin (g/dL)	9.2 (2.1)	9.2 (2.0)
Hematocrit (%)	26.9 (6.1)	27.1 (5.7)
LDH (ratio)^a^	9.2 (4.4)	9.7 (4.7)
Sodium (mEq/l)	131.7 (4.7)	132.2 (5.2)
Potassium (mEq/l)	4.3 (0.8)	4.2 (0.6)
Bicarbonate (mEq/l)	15.8 (3.9)	15.9 (4.4)
pH	7.3 (0.1)	7.3 (0.1)
Neurological involvement, *n* (%)	12 (23)	12 (23)
Somnolence	7 (14)	10 (19)
Seizures	6 (12)	6 (12)
Cardiovascular involvement, *n* (%)	3 (6)	2 (4)
Hemodynamic instability	1 (2)	2 (4)
Tachycardia	2 (4)	0 (0.0)
Gastrointestinal involvement, *n* (%)	40 (77)	31 (60)
Hemorrhagic colitis	3 (6)	2 (4)
Ischemic colitis	1 (2)	0 (0.0)
Increased liver enzymes^b^	40/48 (83)	31/43 (72)
Ileus	2 (4)	0 (0.0)
Gastrointestinal wall thickening	0 (0.0)	1 (2)
Increased pancreatic enzymes	0 (0.0)	1 (2)
Rectal prolapse	3 (6)	2 (4)
Respiratory involvement, *n* (%)	4 (8)	2 (4)
Pulmonary edema	0 (0)	1 (2)
Invasive mechanical ventilation	2 (4)	1 (2)
Tachypnoea	2 (4)	0 (0.0)
Infectious involvement, *n* (%)	1 (2)	0 (0.0)
Bacteremia	1 (2)	0 (0.0)

^a^Lactate dehydrogenase (LDH) values were standardized according to the upper limit of normal of each center (LDH value/LDH upper limit of normal). ^b^Percentages calculated on the evaluable patients (patients on whom liver enzymes were measured). *SD*, standard deviation; *Q1*, first quartile; *Q3*, third quartile; *eGFR*, estimated glomerular filtration rate; *LDH*, lactate dehydrogenase

All subjects except one complied with the assigned doses. This patient received a dose 5% less than expected due to an error in dose calculation. For both SP and MP, there was a mean (SD) of 0.7 (0.5) days between STEC-HUS diagnosis at the participating center and the first dose and a mean (SD) of 5.6 (2.1) days between the start of diarrhea and the first dose. Therapeutic management of prodromal symptoms is shown in Table 1.

For the MP, on day 0, 45 (87%) and 49 (94%) patients presented the STEC-HUS triad in the treatment and control arm, respectively. Seven patients developed the complete triad over the first 3 days after diagnosis. Overall, 49 (94%) patients of the treatment arm and 52 (100%) patients of the control arm developed the complete triad, during the study. The three patients without thrombocytopenia presented minimum platelet counts near the lower limit of normal. Forty-six (89%) and 44 (85%) patients had microbiological confirmation of STEC infection performed by local testing in the treatment and control arm, with serotype O157 being the most frequently detected, followed by O145 (Supplementary Table 4). Genotype *stx*_2_ was the most frequently detected (Supplementary Table 4). After the determination of IgM by the central laboratory, microbiological confirmation reached 96% in the treatment arm.

### Safety outcomes

#### Adverse events (AE)

One hundred and three AE were reported in 38 of the 57 enrolled patients (Table 2; Supplementary Table 5). No AE resulted in study discontinuation, premature discontinuation of the infusion, or precluded administration of the second dose. In two cases, a reduction in infusion rate of the first dose was required due to the presence of hypotension in one case and vomiting in the other. In the first case, the patient had hemodynamic instability with inotropic requirement prior to INM004 infusion, and STEC-HUS was considered as an alternative cause for the hypotension event. In the second case, the patient already presented vomiting prior to the infusion, and STEC-HUS was also considered an alternative explanation for the vomiting during the infusion.

**Table 2:** Overview of adverse events that occurred during the study

Adverse events	Safety population (*n* = 57)
Total number of adverse events	103
Subjects with any adverse, *n* (%)	38 (67)
Number of SAE	4
Subjects with any SAE, *n* (%)	4 (7)
Number of related adverse events	8
Subjects with any related adverse event, *n* (%)	7 (12)
Number of AESI	10
Subjects with any AESI, *n* (%)	6 (11)
Number of adverse events with fatal outcome	0
Subjects with any adverse event with fatal outcome, *n* (%)	0 (0.0)

*SAE*, serious adverse event; *AESI*, adverse event of special interest

Four AE were classified as serious (Supplementary Table 6), and ten were AESI during the study (Supplementary Table 7). None of these events were considered related to INM004, except for one event of rash scarlatiniform (an AESI) assessed as possibly related to INM004, but which could also have been explained by a concomitant COVID infection during hospitalization. There were no cases of anaphylaxis or serum sickness. Of the total AE, eight were considered as possibly related to INM004 by the investigator, none of which was severe or serious, and seven had alternative possible causes (STEC-HUS, other medical conditions, and concomitant medications) (Supplementary Table 8). The DSMB concluded that INM004 showed an adequate safety profile.

#### Laboratory, vital signs, and electrocardiographic findings

No clinically significant abnormalities were found in the laboratory parameters, ECG, or vital signs after the first or second dose of INM004 administration compared to pre-administration of INM004 first dose. None of the patients presented QT/QTc interval prolongation after INM004 administration.

#### PK outcomes

The median (range) *C*_max_ was 56,661.4 (46,399.4–66,923.5) ng/ml for the one-dose regimen. For patients receiving two doses, median (range) *C*_max_ was 41,392.0 (24,086.8–59,538.9) ng/ml after the first dose and 58,298.3 (30,724.4–84,217.4) ng/ml after the second dose. This increase in *C*_max_ after the second dose would be explained for the principle of superposition [21], excluding accumulation due to decreased GFR. Median (range) *t*_1/2_ was 40.6 (31.5–75.1) hours and 56.4 (40.1–72.6) hours for two- and one-dose regimens, respectively. All PK parameters are summarized in Supplementary Table 9. Plasma concentration of INM004 as a function of time for both dose regimens is shown in Supplementary Fig. 1.

### Efficacy outcomes

#### Primary efficacy outcome

The median number of dialysis days was four in the treatment arm and six in the control arm (Table 3). This non-statistically significant difference of 2 days was also observed when only patients who received dialysis were considered (Table 3).

**Table 3:** Efficacy outcomes—matched population

Efficacy outcomes	Treatment arm (*n* = 52)	Control arm (*n* = 52)	*p*-value
Dialysis requirement, *n* (%)	29 (56)	35 (67)	0.23
Dialysis days in all patients^a^, median (Q1:Q3)	4.0 (0.0:9.0)	6.0 (0.0:11.5)	0.43
Dialysis days only in patients who required dialysis, median (Q1:Q3)	8.0 (5.0:16.0)	10.0 (6.0:14.0)	0.95
Dialysis duration in classes, *n* (%)			0.44
0 days	23 (44)	17 (33)	-
1–10 days	19 (37)	21 (40)	-
> 10 days	10 (19)	14 (27)	-
Dialysis duration dichotomic, *n* (%)			0.35
≤ 10 days	42 (81)	38 (73)	-
> 10 days	10 (19)	14 (27)	-
Dialysis requirement after day 0^b^, *n* (%)	15 (40)	23 (58)	0.11
Anuria, *n* (%)	25 (48)	31 (60)	0.24
Anuria days in all patients, median (Q1:Q3)	9.0 (6.0:12.0)	9.0 (6.0:12.0)	0.88
Anuria after day 0^b^, *n* (%)	6 (18)	9 (30)	0.27
Hypertension with medication requirement on day 28, *n* (%)	6 (14)	7 (17)	0.81
Hospitalization days, median (Q1:Q3)	11.0 (7.0:18.5)	13.5 (6.0:19.5)	0.93
Hematuria on day 28^c^, *n* (%)			
Detected	14 (27)	1 (2)	-
Not detected	25 (48)	3 (6)	-
Missing	13 (25)	48 (92)	-
Proteinuria at day 28^c^, *n* (%)			
Detected	18 (35)	3 (6)	-
Not detected	24 (46)	3 (6)	-
Missing	10 (19)	46 (89)	-

^a^For patients without dialysis during the study, a value of zero dialysis day was imputed. ^b^Day 0 is the day of diagnosis of Shiga toxin-producing *Escherichia coli*-associated hemolytic uremic syndrome (STEC-HUS) in the participating site. ^c^Results were not compared between arms due to the high number of missing values in the control arm. *Q1*, first quartile; *Q3*, third quartile

#### Secondary efficacy outcomes

There was a non-statistically significant trend towards a lower number of patients needing dialysis in the treatment compared with the control arm (RR = 0.83; 95% CI 0.61–1.13) (Table 3). Similar non-statistically significant trends were observed for the need for dialysis for more than 10 days (RR = 0.69; 95% CI 0.43–1.10), the requirement of dialysis after day 0 (RR = 0.71; 95% CI 0.35–1.46), the occurrence of anuria (RR = 0.81; 95% CI 0.56–1.16), and the onset of anuria after day 0 (RR = 0.61; 95% CI 0.25–1.50) (Table 3).

**Fig. 4:**
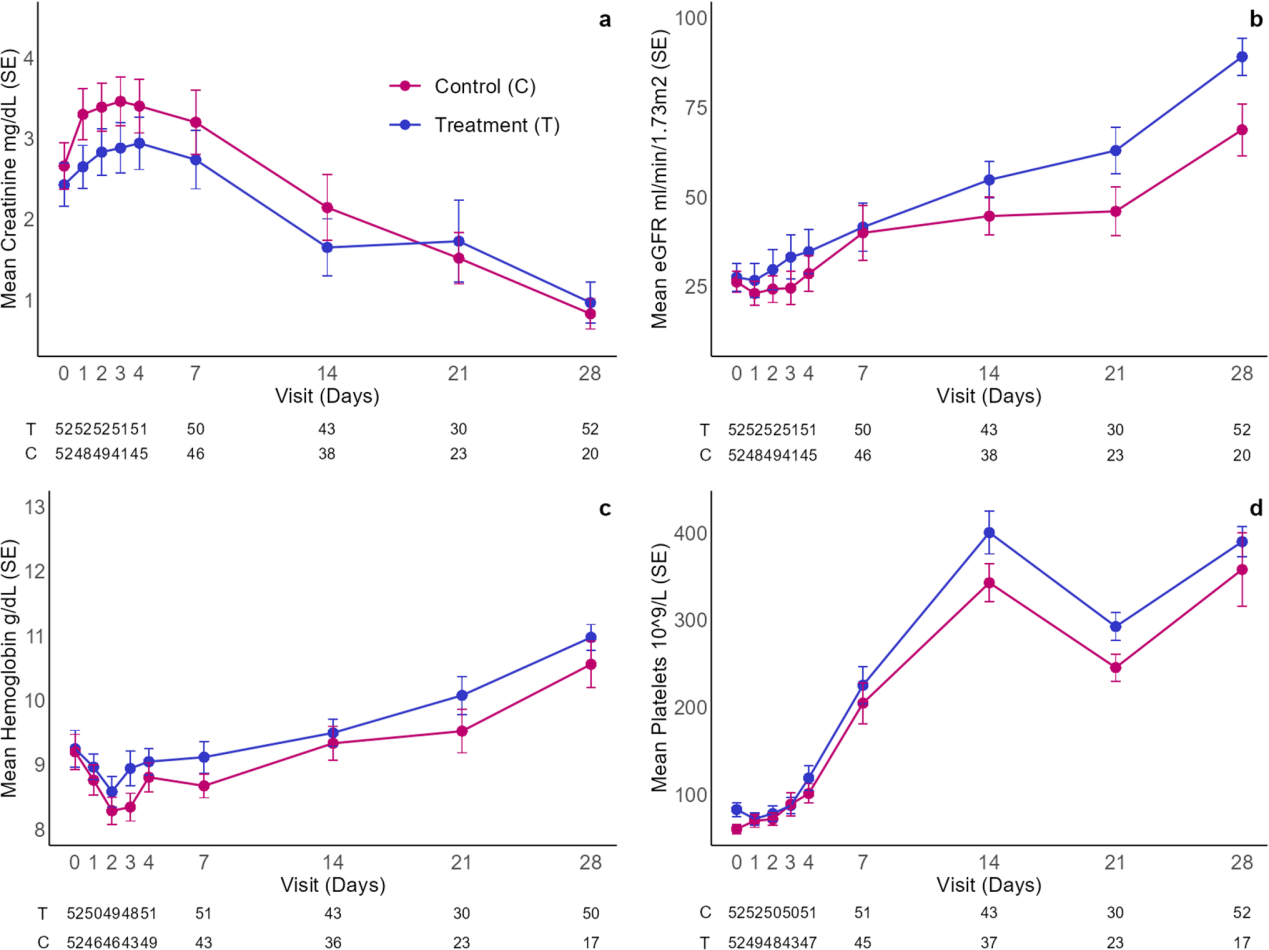
Laboratory parameters of the thrombotic microangiopathy during the entire follow-up period—matched population. **a** Creatinine;** b** eGFR, estimated glomerular filtration rate; **c** hemoglobin; **d** platelets.

**Fig. 5:**
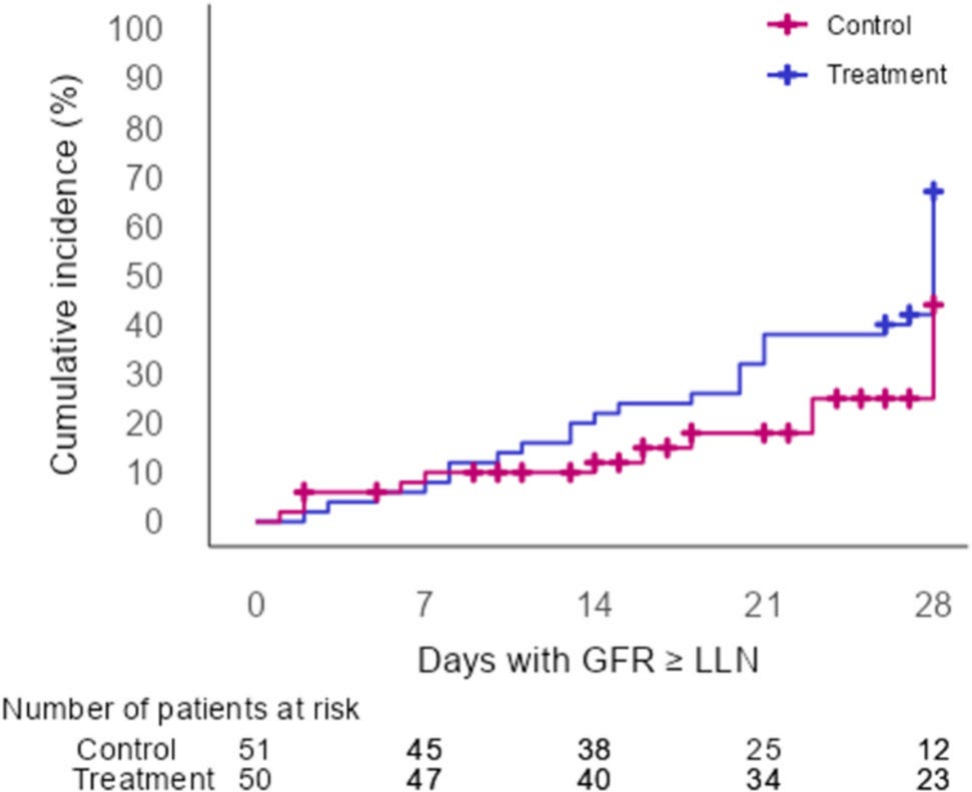
Kaplan–Meier cumulative incidence of glomerular filtration rate recovery during the follow-up period. GFR, glomerular filtration rate; LLN, lower limit of normal

The evolution of creatinine showed a similar pattern in both arms, with values that were overall lower in the treatment arm (Fig. 4, Supplementary Table 10). eGFR showed an increase during the follow-up period in both arms, which started on day 1 and day 3 for the treatment and control arms, respectively, and was overall higher in the treatment arm (Fig. 4, Supplementary Table 10). When the time to kidney function recovery was compared between arms, the treatment arm showed a non-statistically significant trend toward a shorter time to recovery (HR = 1.83; 95% CI 0.95–3.50) (Fig. 5). No differences were observed in the number of patients in each group who presented hypertension requiring medication on day 28 (Table 3). The presence of hematuria and proteinuria at day 28 is presented in Table 3. No comparisons between arms were made for these variables due to the high number of missing data in the control arm.

All patients in both groups achieved platelet counts > 150 × 10^9^/L during the study follow-up period (Fig. 4; Supplementary Table 10), with a median time to recovery of 7 (95% CI 7.0–8.0) days for the treatment group and 9 (95% CI: 8.0–11.0) days for the control group (HR = 1.26; 95% CI 0.84–1.88). The evolution of hemoglobin is shown in Fig. 4 and Supplementary Table 10. No differences between arms were observed in LDH values during the study (Supplementary Table 10). A similar number of patients required red blood cell transfusions (89% and 87% in the treatment and control arms, respectively) (Supplementary Table 11).

A low incidence of neurological, cardiovascular, and gastrointestinal events was observed in both arms after day 0 (Supplementary Table 11). No significant differences were observed in the occurrence of severe extrarenal events (convulsions, cerebral infarction, coma, hemodynamic instability, myocardial failure, myocarditis, hemorrhagic colitis, ischemic colitis, pancreatitis, respiratory distress syndrome, or requirement of mechanical ventilatory assistance for more than 24 h), with eight patients in the treatment arm and ten in the control arm presenting at least one of these events after day 0 (Supplementary Table 12). No differences between arms were observed in hospitalization length (Table 3). No patients in the treatment arm died, and one death was recorded in the control arm.

#### Sensitivity analyses

A similar non-statistically significant trend to that of the primary analysis was observed in time to kidney function recovery in the analysis performed with multiple imputations (HR = 1.71; 95% CI 0.86–3.39).

### Immunogenicity

On day 28, 6 patients exhibited low reactivity. After incubation with INM004, 4 participants exhibited low unspecific reactivity and 2 were non-reactive. Therefore, none of the 57 treated patients showed specific reactivity against INM004.

## Discussion

This phase 2 study aimed to explore the safety, PK profile, and efficacy of INM004, a new therapy for the treatment of STEC-HUS. Its results were expected to provide the first insights on the use of INM004 in pediatric patients with STEC-HUS and to serve as the basis for the design of the phase 3 study.

The study showed that INM004 is a safe therapy for STEC-HUS affected children. INM004 infusions were well tolerated with an adequate benefit/risk profile. There was no evidence of site injection reactions, anaphylaxis, or other severe hypersensitivity reactions associated with INM004 administration. None of the subjects developed symptoms compatible with serum sickness, and no immunogenicity was detected in any of the 57 treated subjects. Overall, safety and immunogenicity findings were consistent with those of phase 1 [16]. Previously, Inmunova had tested similar equine F(ab´)_2_ fragments anti-SARS-Cov-2-RBD (CoviFab®) that showed a safety profile as good as that reported in the present study [22, 23]. Regarding the long-term safety profile, INM004 is given in a total period of 24 h, with a *t*_1/2_ of less than 72 h and no significant accumulation, and its mechanism of action is the specific binding to Stx without interaction with endogenous pathways or binding to human tissues [24]. Therefore, long-term adverse reactions are unlikely. However, standard continuous safety monitoring remains appropriate to detect any unexpected AE.

In the PK sub-study, INM004 showed a profile similar to the one expected, according to the simulated values for pediatric patients with different weights obtained from an allometric calculation based on data from healthy adult volunteers from phase 1. Two doses of INM004 4 mg/kg separated by 24 h showed adequate INM004 blood concentrations during 5 days without significant accumulation despite kidney impairment, as expected for molecules that are not eliminated by renal clearance.

Regarding efficacy, this study showed non-statistically significant trends toward a beneficial effect in several efficacy outcomes related to kidney involvement, including dialysis requirement. These findings might be interpreted as a potential effect of INM004 in the prevention of dialysis or prolonged dialysis. Moreover, a shorter time to kidney function recovery (measured through eGFR recovery) was observed in the treatment compared to the control arm. Altogether, these results could be considered as evidence of less kidney injury in patients treated with INM004. Data from observational studies have shown that the requirement of dialysis and its duration, as well as the presence and duration of anuria, are predictors of kidney sequelae in patients with STEC-HUS [25–28]. Additionally, several studies conducted in pediatric and adult populations with AKI showed that its severity and duration are predictors of evolution to CKD [29–32]. During the early stage of STEC-HUS, circulating Stx is expected to still be present in the bloodstream. Therefore, even when the disease cannot be prevented, it is hypothesized that Stx neutralization by INM004 can ameliorate kidney injury and reduce acute and long-term morbidity.

Regarding extrarenal outcomes, no clinical effect was observed in the present study in hematological parameters. The absence of differences between arms could have several explanations. One possibility is the continuous renewal process of blood components, particularly in STEC-HUS where platelet consumption and mechanical hemolysis occur without bone marrow compromise. This enables a faster recovery of blood components, independent of the slower recovery seen in organs such as the kidneys. Additionally, other mechanisms, not mediated by Stx and Gb3 receptors, may contribute to hemolytic anemia and thrombocytopenia in STEC-HUS, which could explain the reduced impact of INM004 on these parameters [33–35]. Similarly, no differences were observed in the incidence of severe clinical events affecting other target organs, but those were of low incidence. When present, most extrarenal events were ongoing on the day of STEC-HUS diagnosis before INM004 administration.

The main limitation of this study was the non-randomized design, which allowed for the treatment of a considerable number of patients in a short period, but it could have also been a source of bias. To minimize selection bias, measures were adopted in both the study design (same centers, same seasonal period for enrollment, same eligibility criteria, and same SoC for the treatment and the control arm; systematic review of clinical registers for the inclusion of subjects of the control arm) and the statistical analysis (propensity score matching procedure). To address the potential bias introduced by the unblinded design, objective variables were selected as efficacy endpoints. Due to the possibility of attrition bias, as data of the control arm were limited to clinical chart reports, a sensitivity analysis with imputation of missing data was performed for time-to-kidney function recovery. Results were concordant with those of the primary analysis. Additionally, the presence of a higher number of patients with prodromal symptoms and more patients having received antibiotics for the treatment of the prodromal phase in the treatment arm could be explained by an under-registration of these variables in the control arm.

Another limitation was related to the time of the intervention with INM004. Due to its proposed mechanism of action, the prodromic phase (i.e., patients with STEC associated diarrhea) might be the optimal moment to initiate treatment with this product. However, in Argentina, most patients with diarrhea caused by STEC attend health centers of low complexity or are ambulatory managed. Patients are hospitalized at highly complex centers when HUS is already established. For this reason, this study was planned to assess the potential therapeutic effect of INM004 in patients as soon as possible when diagnosis of STEC-HUS was established, with at least two of the three components of the HUS triad.

Regarding the PK profile of INM004, the present study provided adequate and relevant information. However, most of the patients studied were under 4 years old, and the results might not be generalizable to the entire pediatric population. This limitation should be addressed in a phase 3 study, in which the characterization of INM004 is planned to be continued. Finally, as enrollment was lower than expected, it is possible that some potential AE of low incidence (below 1.5%) were not detected. The enrolled sample of 57 subjects yielded a power of 58% to detect AE with this low frequency. As a phase 3 trial with a larger sample size is planned as part of the clinical development of INM004, it will allow us to identify additional events in the case they occur.

In conclusion, this study showed that INM004 can be safely administered to pediatric patients with STEC-HUS and allowed the obtention of PK data that confirmed that the planned regimen of 2 doses of 4 mg/kg is adequate in this population. The study also showed non-statistically significant trends regarding the amelioration of kidney impairment during the acute phase of STEC-HUS. The presented results would allow us to move forward to a phase 3 study to evaluate the efficacy of INM004 in the treatment of STEC-HUS.

## Supplementary Information

Below is the link to the electronic supplementary material.

Graphical abstract (PPTX 116 KB)

Supplementary file2 (PDF 504 KB)

## Data Availability

Datasets are not available because of legal restrictions due to data protection. A request for access can be sent to the corresponding author.
