# Supplementary material for: Open-label, controlled, phase 2 clinical trial assessing the safety, efficacy, and pharmacokinetics of INM004 in pediatric patients with Shiga toxin-producing Escherichia coli–associated hemolytic uremic syndrome
Source: Pediatr Nephrol. 2024 Nov 12;40(6):1983–95. doi: 10.1007/s00467-024-06583-3 (PMC12031759; doi:10.1007/s00467-024-06583-3)
Supplement: Supplementary file 1 — Graphical abstract (PPTX 116 KB) [file 467_2024_6583_MOESM1_ESM.pptx]

## Slide 1
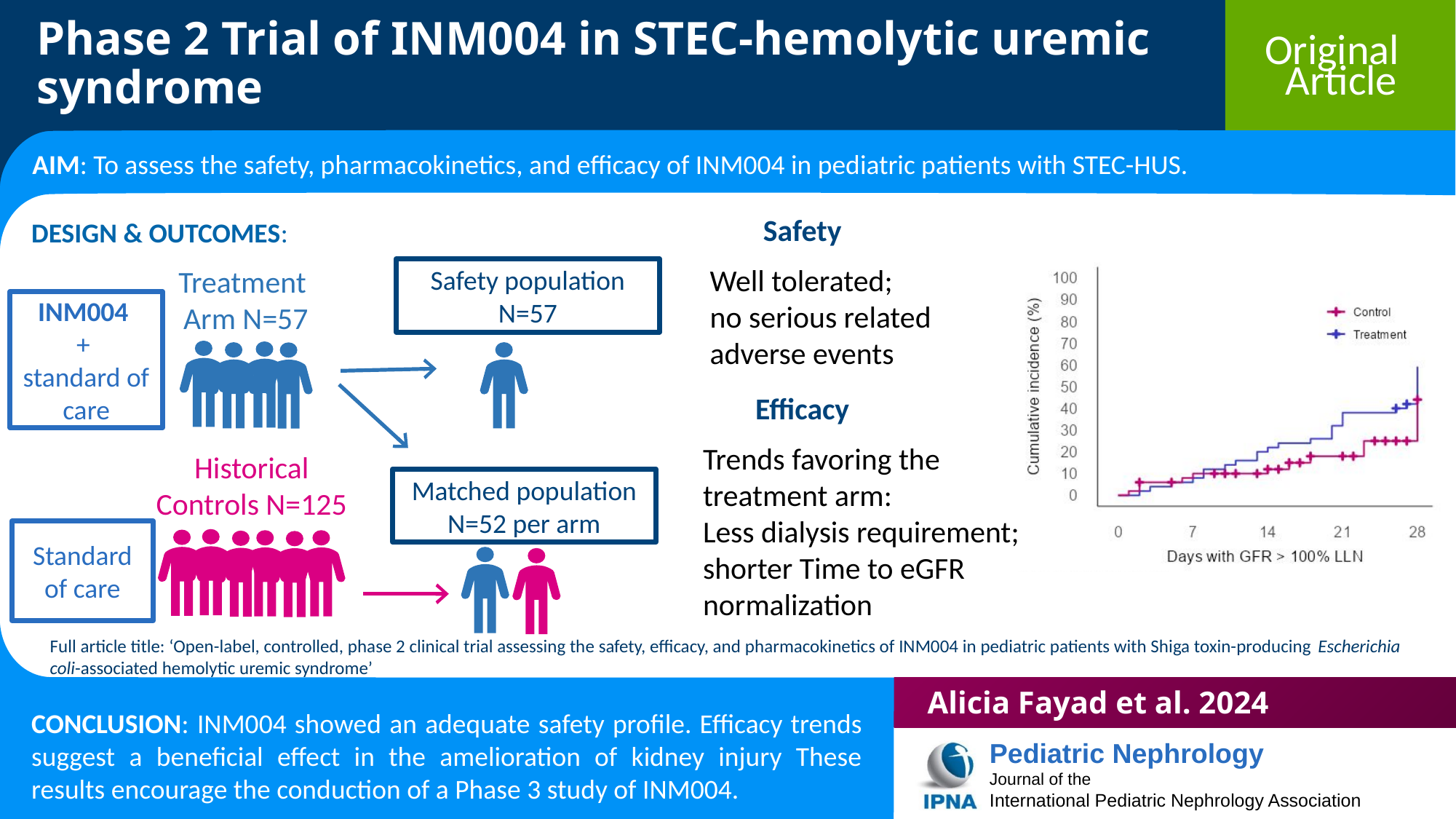

Phase 2 Trial of INM004 in STEC-hemolytic uremic syndrome
AIM: To assess the safety, pharmacokinetics, and efficacy of INM004 in pediatric patients with STEC-HUS.
Safety
DESIGN & OUTCOMES:
Well tolerated;
no serious related
adverse events
Treatment
Arm N=57
Safety population N=57
INM004
+
standard of care
Efficacy
Trends favoring the treatment arm:
Less dialysis requirement;
shorter Time to eGFR normalization
Historical Controls N=125
Matched population N=52 per arm
Standard of care
Full article title: ‘Open-label, controlled, phase 2 clinical trial assessing the safety, efficacy, and pharmacokinetics of INM004 in pediatric patients with Shiga toxin-producing Escherichia coli-associated hemolytic uremic syndrome’
Alicia Fayad et al. 2024
CONCLUSION: INM004 showed an adequate safety profile. Efficacy trends suggest a beneficial effect in the amelioration of kidney injury These results encourage the conduction of a Phase 3 study of INM004.
