# Supplementary material for: Open-label, controlled, phase 2 clinical trial assessing the safety, efficacy, and pharmacokinetics of INM004 in pediatric patients with Shiga toxin-producing Escherichia coli–associated hemolytic uremic syndrome
Source: Pediatr Nephrol. 2024 Nov 12;40(6):1983–95. doi: 10.1007/s00467-024-06583-3 (PMC12031759; doi:10.1007/s00467-024-06583-3)
Supplement: Supplementary file 2 — Supplementary file2 (PDF 504 KB) [file 467_2024_6583_MOESM2_ESM.pdf]

Title: Open-label, controlled, phase 2 clinical trial assessing the safety, efficacy, and pharmacokinetics of INM004 in pediatric patients with Shiga toxin-producing *Escherichia coli*-associated hemolytic uremic syndrome

Journal: Pediatric Nephrology

Authors: Alicia Fayad[1]*; Iliana Principi[2]*; Alejandro Balestracci[3]; Laura Alconcher[4]; Paula Coccia[5]; Marta Adragna[6]; Oscar Amoreo[7]; María Carolina Bettendorff[8]; María Valeria Blumetti[9]; Pablo Bonany[10]; María Laura Flores Tonfi[11]; Luis Flynn[12]; Lidia Ghezzi[5]; Jorge Montero[13]; Flavia Ramírez[14]; Claudia Seminara[15]; Ángela Suarez[16]; Ana Paula Spizzirri[16]; Marta Rivas[17]; Mariana Pichel[17]; Vanesa Zylberman[17]; Linus Spatz[17]; Carolina Massa[17]; Marina Valerio[17]; Santiago Sanguineti[17]; Mariana Colonna[17]; Ian Roubicek[17]; Fernando Goldbaum[17,18]; on behalf of INM004 Study Group[19].

[1]Hospital de Niños Ricardo Gutiérrez, Ciudad Autónoma de Buenos Aires, Argentina

[2]Hospital Pediátrico Dr. Humberto J. Notti, Mendoza, Argentina

[3]Hospital General de Niños Pedro de Elizalde, Ciudad Autónoma de Buenos Aires, Argentina

[4]Hospital Interzonal Regional Dr. José Penna, Buenos Aires, Argentina

[5]Hospital Italiano de Buenos Aires, Ciudad Autónoma de Buenos Aires, Argentina

[6]Hospital de Pediatría S.A.M.I.C. Prof. Dr. Juan P. Garrahan, Ciudad Autónoma de Buenos Aires, Argentina

[7]Hospital de Alta Complejidad en Red El Cruce Dr. Néstor Carlos Kirchner, Buenos Aires, Argentina

[8]Sanatorio Allende, Córdoba, Argentina

[9]Clínica Zabala, Ciudad Autónoma de Buenos Aires, Argentinax|

[10]Establecimiento Asistencial Dr. Lucio Molas, Santa Rosa, Argentina

[11]Sanatorio Güemes

[12]Sanatorio de Niños, Rosario, Argentina

[13]Hospital Interzonal Especializado Materno Infantil Don Victorio Tetamanti, Buenos Aires, Argentina

[14]Hospital Provincial Neuquén, Dr. Eduardo Castro Rendón, Neuquén, Argentina

[15]Hospital de Niños de la Santísima Trinidad, Córdoba, Argentina

[16]Hospital de Niños Sor María Ludovica, Buenos Aires, Argentina

[17]Inmunova SA, Ciudad Autónoma de Buenos Aires, Argentina

[18]National Scientific and Technological Research Council, CONICET, Ciudad Autónoma de Buenos Aires, Argentina

[19]Additional members of this group are listed in Supplementary Appendix.

* These authors contributed equally

Corresponding Author: Fernando Goldbaum

- Email: fgoldbaum@inmunova.com

INM004 Study group

José Miguel Liern[1]; Gimena Ramírez[1]; Mariana Kamariski[2]; Pablo Javier[2]; Julián Gonzalez[2]; Luciana Lavado[2]; Mariana Luna[2]; Ismael Toledo[3]; Lucas Ivan Lucarelli[4]; Sabrina Bronfen[4]; Juliana Blazquez[5]; Verónica Ferraris[5]; Claudia Raddavero[5]; Alicia Chaparro[6]; Rosa Bologna[6]; Juan Ibañez[6]; Fabrizio Locane[6]; Mariana Langard[7]; Paula Bresso[7]; Alejo de Sarasqueta[7]; Soledad Bergallo[8]; Nicolás Gonzalez[9]; Rodrigo Gonzalez[9]; Mariangeles Gil[9]; Edgardo Bazano[9]; Natali Leimann[9]; María Alejandra Mortarini[9]; Hilda Susana Perez[10]; Tatiana Bressan[10]; Pablo Alberto Bertolone[10]; Lautaro Del Greco[10]; Isis Perez[10]; Carola Abilar[10]; Jorge Alejandro Brigante[11]; Cecilia Córdoba[11]; María Griselda Gutiérrez[12]; Marilina Viñuela[12]; Patricia María Rivichini[12]; Silvia Siacaluga[12]; Cintia Larrubia[13]; Agustina Garcia Pósleman[13]; Guillermo Monaldi[13]; Melisa Ruiz Diaz[13]; Tatiana Barrionuevo[14]; Paula Rossi[14]; Romina Brandolisio[14]; Veronica Selva[15]; Mirian Edith Morales[15]; María Florencia Mieres Estigarribia[15]; Emanuel De Rose[16]; Laura Lombardi[16]; Priscila Pereyra[16]; María José Gogorza[16]

[1]Hospital de Niños Ricardo Gutiérrez, Ciudad Autónoma de Buenos Aires, Argentina

[2]Hospital Pediátrico Dr. Humberto J. Notti, Mendoza, Argentina

[3]Hospital General de Niños Pedro de Elizalde, Ciudad Autónoma de Buenos Aires, Argentina

[4]Hospital Interzonal Regional Dr. José Penna, Buenos Aires, Argentina

[5]Hospital Italiano de Buenos Aires, Ciudad Autónoma de Buenos Aires, Argentina

[6]Hospital de Pediatría S.A.M.I.C. Prof. Dr. Juan P. Garrahan, Ciudad Autónoma de Buenos Aires, Argentina

[7]Hospital de Alta Complejidad en Red El Cruce Dr. Néstor Carlos Kirchner, Buenos Aires, Argentina

[8]Sanatorio Allende, Córdoba, Argentina

[9]Clínica Zabala, Ciudad Autónoma de Buenos Aires, Argentina|

[10]Establecimiento Asistencial Dr. Lucio Molas, Santa Rosa, Argentina

[11]Sanatorio Güemes

[12]Sanatorio de Niños, Rosario, Argentina

[13]Hospital Interzonal Especializado Materno Infantil Don Victorio Tetamanti, Buenos Aires, Argentina

[14]Hospital Provincial Neuquén, Dr. Eduardo Castro Rendón, Neuquén, Argentina

[15]Hospital de Niños de la Santísima Trinidad, Córdoba, Argentina

[16]Hospital de Niños Sor María Ludovica, Buenos Aires, Argentina

Supplementary Table 1. Demographic and baseline clinical characteristics of the Safety Population

Demographic and baseline characteristics	Safety population (n=57)
Age (years), *mean (SD)*	2.6 (1.9)
Female sex, *n (%)*	27 (47)
Weigh (kg), *mean (SD)*	15.1 (6.5)
Height (cm), *mean (SD)*	95.0 (14.7)
BMI (kg/m^2), *mean (SD)*	16.2 (2.3)
Prodromal Symptoms, *n (%)*	
Diarrhea	57 (100)
Bloody diarrhea	44 (77)
Abdominal pain	39 (68)
Fever	28 (49)
Vomiting	45 (79)
Days from diarrhea onset to diagnosis, *Median (Q1:Q3)*	4.9 (2.3)
Vital signs, *mean (SD)*	
Respiratory rate (breaths/min)	28.5 (7.9)
Pulse rate (beats/min)	122.1 (24.6)
Systolic blood pressure (mmHg)	106.5 (18.8)
Diastolic blood pressure (mmHg)	64.3 (16.1)
Temperature (ºC)	36.4 (0.7)
Baseline laboratory parameters, *mean (SD)*	
Creatinine (mg/dL)	2.7 (2.3)
eGFR (ml/min/1.73 m^2)	26.1 (27.4)
Urea (mg/dL)	127.9 (78.7)
Leukocytes (10^9/l)	18.3 (9.1)
Neutrophils (%)	59.2 (11.2)
Platelets (10^9/l)	82.9 (55.8)
Hemoglobin (g/dL)	9.2 (2.0)
Hematocrit (%)	26.9 (6.0)
LDH (ratio)[a]	9.5 (4.5)
Sodium (mEq/l)	131.7 (4.8)
Potassium (mEq/l)	4.2 (0.8)
Bicarbonate (mEq/l)	15.9 (3.8)
pH	7.3 (0.1)

[a]Lactate dehydrogenase (LDH) values were standardized according to the upper limit of normal of each center (LDH value / LDH upper limit of normal).

SD, standard deviation; *BMI*, body mass index; *LDH*, lactate dehydrogenase; *eGFR*, estimated glomerular filtration rate

Supplementary Table 2. Demographic and baseline clinical characteristics of the Full Analysis Set (FAS)

Demographic and baseline characteristics	Treatment arm (n=57)	Control arm (n=125)
Age (years), *mean (SD)*	2.6 (1.9)	3.0 (2.2)
Female sex, *n (%)*	27 (47)	67 (54)
Weight (kg), *mean (SD)*	15.1 (6.5)	15.4 (6.1)
Height (cm), *mean (SD)*	95.0 (14.7)	96.5 (16.5)
BMI (kg/m2), *mean (SD)*	16.2 (2.3)	16.1 (2.2)
Prodromal Symptoms, *n (%)*		
Non-bloody diarrhea	57 (100)	125 (100)
Bloody diarrhea	44 (77)	97 (78)
Abdominal pain	39 (69)	50 (40)
Fever	28 (49)	45 (36)
Vomiting	45 (79)	83 (66)
Therapeutic management of prodromal symptoms, *n (%)*		
Pre-admission antibiotics	12 (21)	23 (18)
Expansion	23 (40)	52 (42)
Days from diarrhea onset to diagnosis, *Median (Q1:Q3)*	4.9 (2.3)	5.0 (2.3)
Baseline laboratory parameters, *mean (SD)*		
Creatinine (mg/dL)	2.7 (2.3)	2.5 (2.2)
eGFR (ml/min/1.73 m2)	26.1 (27.4)	30.4 (26.9)
Urea (mg/dL)	127.9 (78.7)	142.0 (102.2)
Leukocytes (10^9/l)	18.3 (9.1)	18.3 (10.0)
Neutrophils (%)	59.2 (11.2)	60.0 (12.7)
Platelets (10^9/l)	82.9 (55.8)	70.4 (54.0)
Hemoglobin (g/dL)	9.2 (2.0)	9.3 (1.9)
Hematocrit (%)	26.9 (6.0)	27.0 (5.5)
LDH (ratio)	9.5 (4.5)	9.5 (5.9)
Sodium (mEq/l)	131.7 (4.8)	132.2 (5.4)
Potassium (mEq/l)	4.2 (0.8)	4.2 (0.7)
Bicarbonate (mEq/l)	15.9 (3.8)	16.2 (4.7)
pH	7.3 (0.1)	7.3 (0.1)
Neurological involvement, *n (%)*	12 (21)	25 (20)
Somnolence	7 (12)	20 (16)
Stupor	0 (0.0)	1 (0.8)
Seizures	6 (11)	9 (7)

Demographic and baseline characteristics	Treatment arm (n=57)	Control arm (n=125)
Other non-pre-specified	1 (2)	0 (0.0)
Cardiovascular involvement, *n (%)*	3 (5)	5 (4)
Hemodynamic instability	1 (2)	5 (4)
Arrhythmia	0 (0.0)	1 (0.8)
Other non-pre-specified	2 (4)	0 (0.0)
Gastrointestinal involvement, *n (%)*	43 (75)	73 (58)
Hemorrhagic colitis	3 (5)	5 (4)
Ischemic colitis	1 (2)	0 (0.0)
Increased liver enzymes[a]	43/51 (84)	70/100 (70)
Ilium	2 (4)	0 (0.0)
Pancreatitis	0 (0.0)	1 (0.8)
Other non-pre-specified	3 (5)	9 (7)
Respiratory involvement, *n (%)*	5 (9)	6 (5)
Pulmonary edema	0 (0.0)	2 (1.6)
Respiratory distress syndrome	0 (0.0)	2 (1.6)
Invasive mechanical ventilation	2 (3.5)	3 (2.4)
Other non-pre-specified	3 (5.3)	1 (0.8)
Infectious involvement, *n (%)*	1 (1.8)	2 (1.6)
Bacteremia	1 (1.8)	2 (1.6)

[a]Percentages calculated on the evaluable patients (patients on whom liver enzymes were measured)
SD, standard deviation; *Q1*, first quartile; *Q3*, third quartile; *STEC-HUS*, Shiga toxin-producing *Escherichia coli* Hemolytic Uremic Syndrome; *eGFR*, estimated glomerular filtration rate; *LDH*, lactate dehydrogenase

Supplementary Table 3. Demographic data of the subjects included in the PK sub-study

Dose regimen	Gender	Age (years)	Weight (kg)
Two doses	Female	2	21.4
	Female	4	16.8
	Male	1	9.8
	Male	1	9.2
	Male	1	11.8
	Female	1	11.4
	Male	3	15.2
	Male	3	14.8
One dose	Female	7	31.8
	Male	1	10.0

Supplementary Table 4. Microbiological diagnosis of Shiga toxin-producing *Escherichia coli* (STEC) – Matched population

	Treatment arm (n=52)	Control arm (n=52)
Number of participants with STEC-HUS with microbiology confirmation, n (%)	46 (89)	44 (85)
Serotype, n (%)		
0157	27 (52)	24 (46)
0145	8 (15)	7 (14)
Unknown	11 (21)	13 (25)
Genotype, n (%)		
Stx2	22 (42)	23 (44)
Stx2a	3 (6)	5 (10)
Stx2a/2c	3 (6)	9 (17)
Stx2 ND	16 (31)	9 (17)
Stx1/Stx2	3 (6)	0 (0.0)
St1 ND /Stx 2 ND	3 (6)	0 (0.0)
Stx generic	9 (17)	9 (17)
Not detected	12 (23)	12 (23)
Missing, n (%)	0 (0.0)	1 (2)

STEC-HUS, Shiga toxin producing *Escherichia coli*- hemolytic uremic syndrome; *Stx,* Shiga toxin; *ND*, not done

Supplementary Table 5. Adverse events by Primary System Organ Class – Preferred term according to their relationship with INM004. N=57

System Organ Class[a]<br>Preferred Term xx/zz (xx%)[b]	Related	Not related	All
Subjects with any AE	8/7 (12)	95/36 (63)	103/38 (68)
Infections and infestations	0/0 (0.0)	23/19 (33)	23/19 (33)
Pneumonia	0/0 (0.0)	4/4 (7)	4/4 (7)
COVID-19	0/0 (0.0)	3/3 (5)	3/3 (5)
Upper respiratory tract infection	0/0 (0.0)	3/3 (5)	3/3 (5)
Gastroenteritis	0/0 (0.0)	2/2 (4)	2/2 (4)
Abdominal wall infection	0/0 (0.0)	1/1 (2)	1/1 (2)
Bronchitis	0/0 (0.0)	1/1 (2)	1/1 (2)
Infectious pleural effusion	0/0 (0.0)	1/1 (2)	1/1 (2)
Laryngitis	0/0 (0.0)	1/1 (2)	1/1 (2)
Pharyngitis	0/0 (0.0)	1/1 (2)	1/1 (2)
Postoperative wound infection	0/0 (0.0)	1/1 (2)	1/1 (2)
Rhinitis	0/0 (0.0)	1/1 (2)	1/1 (2)
Sepsis	0/0 (0.0)	1/1 (2)	1/1 (2)
Skin infection	0/0 (0.0)	1/1 (2)	1/1 (2)
Tracheitis	0/0 (0.0)	1/1 (2)	1/1 (2)
Urinary tract infection	0/0 (0.0)	1/1 (2)	1/1 (2)
Respiratory, thoracic and mediastinal disorders	0/0 (0.0)	17/14 (25)	17/14 (25)
Pleural effusion	0/0 (0.0)	4/4 (7)	4/4 (7)
Stridor	0/0 (0.0)	3/3 (5)	3/3 (5)
Bronchospasm	0/0 (0.0)	2/2 (4)	2/2 (4)
Atelectasis	0/0 (0.0)	1/1 (2)	1/1 (2)
Epistaxis	0/0 (0.0)	1/1 (2)	1/1 (2)
Hypoventilation	0/0 (0.0)	1/1 (2)	1/1 (2)
Laryngeal stenosis	0/0 (0.0)	1/1 (2)	1/1 (2)
Respiratory tract haemorrhage	0/0 (0.0)	1/1 (2)	1/1 (2)
Rhinorrhoea	0/0 (0.0)	1/1 (2)	1/1 (2)
Tachypnoea	0/0 (0.0)	1/1 (2)	1/1 (2)
Wheezing	0/0 (0.0)	1/1 (2)	1/1 (2)
Product issues	0/0 (0.0)	11/10 (18)	11/10 (18)
Device malfunction	0/0 (0.0)	11/10 (18)	11/10 (18)
Gastrointestinal disorders	2/2 (4)	8/7 (12)	10/8 (14)
Abdominal pain	0/0 (0.0)	4/4 (7)	4/4 (7)
Vomiting	2/2 (4)	4/3 (5)	6/4 (7)
Skin and subcutaneous tissue disorders	1/1 (12)	11/7 (12)	12/7 (12)
Rash	0/0 (0.0)	5/4 (7)	5/4 (7)
Dermatitis diaper	0/0 (0.0)	1/1 (2)	1/1 (2)

System Organ Class[a]<br>Preferred Term xx/zz (xx%)[b]	Related	Not related	All
Granuloma skin	0/0 (0.0)	1/1 (2)	1/1 (2)
Pruritus	0/0 (0.0)	1/1 (2)	1/1 (2)
Rash scarlatiniform	1/1 (2)	1/1 (2)	2/1 (2)
Skin lesion	0/0 (0.0)	1/1 (2)	1/1 (2)
Urticaria	0/0 (0.0)	1/1 (2)	1/1 (2)
Metabolism and nutrition disorders	0/0 (0.0)	8/6 (11)	8/6 (11)
Hypouricaemia	0/0 (0.0)	3/3 (5)	3/3 (5)
Hypokalaemia	0/0 (0.0)	2/2 (4)	2/2 (4)
Hyperglycaemia	0/0 (0.0)	1/1 (2)	1/1 (2)
Hyponatraemia	0/0 (0.0)	1/1 (2)	1/1 (2)
Metabolic alkalosis	0/0 (0.0)	1/1 (2)	1/1 (2)
General disorders and administration site conditions	0/0 (0.0)	7/5 (9)	7/5 (9)
Pyrexia	0/0 (0.0)	5/4 (7)	5/4 (7)
Catheter site haemorrhage	0/0 (0.0)	1/1 (2)	1/1 (2)
Withdrawal syndrome	0/0 (0.0)	1/1 (2)	1/1 (2)
Investigations	2/2 (4)	2/2 (4)	4/4 (7)
Hepatic enzyme increased	2/2 (4)	0/0 (0.0)	2/2 (4)
Monocyte count decreased	0/0 (0.0)	2/2 (4)	2/2 (4)
Vascular disorders	1/1 (2)	3/3 (5)	4/4 (7)
Hypotension	1/1 (2)	1/1 (2)	2/2 (4)
Haematoma	0/0 (0.0)	1/1 (2)	1/1 (2)
Phlebitis	0/0 (0.0)	1/1 (2)	1/1 (2)
Blood and lymphatic system disorders	2/2 (4)	1/1 (2)	3/3 (5)
Lymphocytosis	2/2 (4)	0/0 (0.0)	2/2 (4)
Neutropenia	0/0 (0.0)	1/1 (2)	1/1 (2)
Injury, poisoning and procedural complications	0/0 (0.0)	3/3 (5)	3/3 (5)
Alcohol poisoning	0/0 (0.0)	1/1 (2)	1/1 (2)
Catheter site haematoma	0/0 (0.0)	1/1 (2)	1/1 (2)
Wrong product administered	0/0 (0.0)	1/1 (2)	1/1 (2)
Hepatobiliary disorders	0/0 (0.0)	1/1 (2)	1/1 (2)
Hepatitis	0/0 (0.0)	1/1 (2)	1/1 (2)

[a]The primary SOC is sorted by the internationally agreed SOC order and PTs ordered by the decreasing frequency within each SOC.
[b]xx/zz (xx%) = number of events / number of subjects with at least one event (percentage of patients with at least one event)
AE, adverse event

Supplementary Table 6. Description of serious adverse events

Adverse event	AESI	Relationship with INM004	Alternative causes	Severity	Narrative
Laryngeal stenosis	No	Unrelated	STEC-HUS, other medical condition	Life-threatening	The event was attributed to iatrogenic injury caused by endotracheal intubation.
Urticaria	Yes	Unrelated	Concomitant medication	Moderate	The event occurred 11 days after the second dose and was attributed to cephalexin treatment for a urinary tract infection.
Gastroenteritis	No	Unrelated	Other medical condition	Moderate	The event required rehospitalization after initial discharge and was attributed to Norovirus infection.
Error in the medication dispensed	No	Unrelated	Concomitant medication	Mild	An error in the dispense of a concomitant medication occurred after patient discharge and required rehospitalization to monitor possible adverse reactions.

AESI, adverse event of special interest; *STEC-HUS*, Shiga toxin-producing *Escherichia coli*-associated-hemolytic uremic syndrome.

Supplementary Table 7. Adverse events of special interest

Adverse event	Serious	Severity	Relationship to INM004	Other possible causes
Rash	No	Mild	Unrelated	STEC-HUS, other medical condition
Rash	No	Mild	Unrelated	Other medical condition
Rash	No	Mild	Unrelated	None
Rash	No	Mild	Unrelated	Concomitant medication
Rash	No	Moderate	Unrelated	Other medical condition
Scarlatiniform rash	No	Moderate	Possibly related	Other medical condition
Scarlatiniform rash	No	Mild	Unrelated	Other medical condition
Urticaria	Yes	Moderate	Unrelated	Concomitant medication
Neutropenia	No	Mild	Unrelated	Other medical condition
Hepatitis	No	Moderate	Unrelated	STEC-HUS

STEC-HUS, Shiga toxin-producing *Escherichia coli*-associated hemolytic uremic syndrome

Supplementary Table 8. Study adverse events classified as possibly related to INM004

Adverse event	AESI	Serious	Other possible causes	Severity
Hypotension	No	No	STEC-HUS	Moderate
Hepatic enzyme increased	No	No	Concomitant medication, STEC-HUS	Mild
Hepatic enzyme increased	No	No	None	Moderate
Lymphocytosis	No	No	STEC-HUS	Mild
Lymphocytosis	No	No	STEC-HUS	Mild
Rash scarlatiniform	Yes	No	Other medical condition	Moderate
Vomiting	No	No	Concomitant medication, STEC-HUS	Mild
Vomiting	No	No	STEC-HUS	Moderate

AE, adverse event, *PT*, preferred term, *AESI*, adverse event of special interest; *STEC-HUS*, Shiga toxin-producing *Escherichia coli*-associated hemolytic uremic syndrome

Supplementary Table 9. Pharmacokinetic parameters of INM004 in one-dose and two-dose regimens

Parameter, *median (range)*	One dose regimen (n=2)	Two dose regimen (n=8)
$Tmax_1$ (h)	0.8 (0.8: 0.9)	0.9 (0.8: 0.9)
$Tmax_2$ (h)	NA	24.5 (0.9: 25.0)
$Cmax_1$ (ng/mL)	56661.4 (46399.4: 66923.5)	41392.0 (24086.8: 59538.9)
$Cmax_2$ (ng/mL)	NA	58298.3 (30724.4: 84217.4)
AUC_{0-t} (h*ng/mL)	1825054.6 (1264461.9: 2385647.3)	2511419.3 (1289087.6: 3765505.7)
AUC_{0-inf} (h*ng/mL)	2238657.6 (1379621.6: 3097693.6)	2779172.2 (1509884.6: 4564721.8)
$T_{1/2}$ (h)	56.4 (40.1: 72.6)	40.6 (31.5: 72.1)
Lambda-z (/h)	0.013 (0.010: 0.017)	0.017 (0.010: 0.022)
Cl (ml/h/kg)	2.1 (1.3: 2.9)	1.4 (0.9: 2.6)
Vd (ml/kg)	151.6 (135.2: 167.9)	84.2 (56.9: 145.7)
C_{max}/D (kg*ng/ml/mg)	14165.4 (11599.8: 16730.9)	14691.5 (7681.1: 21054.4)
AUC_{0-t}/ D (h*kg*ng/ml/mg)	456263.7 (316115.5: 596411.8)	627854.8 (322271.9: 941376.4)
AUC_{0-inf}/ D (h*kg*ng/ml/mg)	559664.4 (344905.4: 774423.4)	694793.0 (377471.1: 1141180.4)

$Tmax_1$, time to peak plasma concentration of INM004 after the first dose; *$Tmax_2$*, time from time 0 to peak plasma concentration of INM004 after the second dose; *$Cmax_1$*, peak plasma concentration of INM004 after the first dose; *$Cmax_2$*, peak plasma concentration of INM004 after the second dose; *AUC_{0-t}*, area under the concentration curve as a function of time, from initial time to terminal time; *AUC_{0-inf}.*, area under the concentration curve as a function of time, from initial time extrapolated to infinity; *T½*, half-life; *Lambda-z*, terminal half-life; *Cl*, clearence; *Vd*, volume of distribution; *D*, dose

Supplementary Table 10. Laboratory parameters of the thrombotic microangiopathy during the study follow-up period – Matched population

Laboratory parameter, *mean (SD)*	Treatment arm (n=52)					Control arm (n=52)				
	Day 0[a]	Min	Max	At Discharge	Day 28	Day 0[a]	Min	Max	At Discharge	Day 28
Hemoglobin (g/dL)	9.2 (2.1)	7.1 (1.3)	11.0 (1.3)	9.4 (1.4)	11.0 (1.4)	9.2 (2.0)	7.0 (1.3)	11.1 (3.7)	9.3 (1.4)	10.6 (1.5)
Missing	0	0	0	0	2	0	0	3	0	35
Platelets (10^9/L)	82.7 (57.9)	52.8 (45.1)	380.6 (157.4)	328.5 (139.3)	388.4 (116.7)	60.3 (37.5)	38.3 (26.8)	296.9 (140.6)	271.2 (131.3)	358.1 (173.3)
Missing	0	0	0	0	0	0	0	1	0	35
LDH (ratio)[b]	9.2 (4.4)	2.9 (1.9)	11.2 (5.9)	3.0 (1.6)	1.4 (0.4)	9.7 (4.7)	3.3 (2.3)	11.7 (5.8)	3.5 (2.9)	1.4 (0.5)
Missing	1	0	0	0	5	8	4	1	4	40
Creatinine (mg/dL)	2.4 (1.9)	0.9 (0.7)	3.9 (2.8)	0.8 (0.5)	1.0 (1.8)	2.7 (2.1)	1.1 (0.8)	4.4 (2.9)	1.0 (0.6)	0.8 (0.8)
Missing	0	0	0	0	0	0	2	0	0	32
eGFR (ml/min/1,73m^2)	27.6 (28.2)	19.6 (26.9)	95.5 (44.5)	69.6 (41.7)	89.2 (37.5)	26.3 (21.3)	18.6 (19.6)	78.9 (42.8)	53.2 (29.5)	68.8 (32.3)
Missing	0	0	0	0	0	0	0	0	0	32

[a]Day 0 corresponds to the day of Shiga toxin-producing *Escherichia coli* associated-hemolytic uremic syndrome (STEC-HUS) diagnosis in the participating site.
[b]Lactate dehydrogenase (LDH) values were standardized according to the upper limit of normal of each center (LDH value / LDH upper limit of normal)
SD, Standard deviation; *Min*, minimum value recorded during hospitalization, *Max*, maximum value recorded during hospitalization; *LDH*, lactate dehydrogenase; *eGFR*, estimated glomerular filtration rate

Supplementary Table 11. Extrarenal involvement and therapeutic interventions during the study follow-up period – Matched population

Extrarenal involvement/ Intervention, *n (%)*	Treatment arm (n=52)			Control arm (n=52)		
	Day 0[a]	Post Day 0[a]	Complete follow-up	Day 0[a]	Post Day 0[a]	Complete follow-up
Neurological involvement	12 (23)	8 (15)	15 (29)	12 (23)	9 (17)	18 (35)
Impaired consciousness	7 (14)	6 (12)	12 (23)	10 (19)	6 (12)	15 (29)
Somnolence	7 (14)	5 (10)	12 (23)	10 (19)	5 (10)	15 (29)
Stupor	0 (0.0)	1 (2)	1 (2)	0 (0.0)	1 (2)	1 (2)
Coma	0 (0.0)	0 (0.0)	0 (0.0)	0 (0.0)	0 (0.0)	0 (0.0)
Seizures	6 (12)	3 (6)	8 (15)	6 (12)	0 (0.0)	6 (12)
Focal	0 (0.0)	2 (4)	2 (4)	0 (0.0)	0 (0.0)	0 (0.0)
Generalized	5 (10)	2 (4)	7 (14)	5 (10)	0 (0.0)	5 (10)
Status epilepticus	1 (2)	1 (2)	2 (4)	1 (2)	0 (0.0)	1 (2)
Focal deficit	0 (0.0)	2 (4)	2 (4)	0 (0.0)	1 (2)	1 (2)
Other non-prespecified	1 (2)	1 (2)	2 (4)	0 (0.0)	2 (4)	2 (4)
Cardiovascular involvement	3 (6)	4 (8)	7 (14)	2 (4)	5 (10)	7 (14)
Hemodynamic instability	1 (2)	3 (6)	4 (8)	2 (4)	4 (8)	6 (12)
Acute myocardial infarction	0 (0.0)	0 (0.0)	0 (0.0)	0 (0.0)	0 (0.0)	0 (0.0)
Arrythmia	0 (0.0)	0 (0.0)	0 (0.0)	0 (0.0)	0 (0.0)	0 (0.0)
Myocarditis	0 (0.0)	0 (0.0)	0 (0.0)	0 (0.0)	0 (0.0)	0 (0.0)
Other non-prespecified	2 (4)	1 (2)	3 (6)	0 (0.0)	1 (2)	1 (2)
Gastrointestinal involvement	40 (77)	14 (27)	43 (83)	31 (60)	15 (29)	41 (80)
Hepatitis/increased liver enzymes	40 (77)	2 (4)	42 (81)	31 (60)	4 (8)	35 (67)
Hemorrhagic colitis	3 (6)	1 (2)	4 (8)	2 (4)	1 (2)	3 (6)
Ischemic colitis	1 (2)	0 (0.0)	1 (2)	0 (0.0)	0 (0.0)	0 (0.0)
Intussusception	0 (0.0)	0 (0.0)	0 (0.0)	0 (0.0)	0 (0.0)	0 (0.0)

Extrarenal involvement/ Intervention, *n (%)*	Treatment arm (n=52)			Control arm (n=52)		
	Day 0[a]	Post Day 0[a]	Complete follow-up	Day 0[a]	Post Day 0[a]	Complete follow-up
Pancreatitis	0 (0.0)	0 (0.0)	0 (0.0)	0 (0.0)	1 (2)	1 (2)
Ilium	2 (4)	1 (2)	3 (6)	0 (0.0)	2 (4)	2 (4)
Other non-prespecified	3 (6)	10 (19)	13 (25)	5 (10)	10 (19)	14 (27)
Endrocrine involvement	1 (2)	0 (0.0)	1 (2)	1 (2)	2 (4)	3 (6)
Hyperglycemia with insulin requirement	0 (0.0)	0 (0.0)	0 (0.0)	0 (0.0)	1 (2)	1 (2)
Other non-prespecified	1 (2)	0 (0.0)	1 (2)	1 (2)	1 (2)	2 (4)
Therapeutic intervention						
Expansion	23 (44)	1 (2)	24 (46)	22 (42)	2 (4)	24 (46)
Mechanical ventilation	2 (4)	6 (12)	8 (15)	1 (2)	4 (8)	5 (10)
Plasmapheresis	0 (0.0)	2 (4)	2 (4)	0 (0.0)	1 (2)	1 (2)
Platelet transfusion	7 (14)	8 (16)	15 (29)	5 (10)	10 (19)	15 (29)
Red blood cells transfusion	17 (33)	29 (56)	46 (89)	14 (27)	31 (60)	45 (87)

[a]Day 0 corresponds to the date of the Shiga toxin-producing *Escherichia coli* associated-hemolytic uremic syndrome (STEC-HUS) diagnosis in the participating site.

Supplementary Table 12. Overview of clinical events associated to severe extrarenal involvements during the study follow-up period – Matched population

Severe extrarenal involvement, *n (%)*	Treatment group (n=52)			Control group (n=52)		
	Day 0[a]	After Day 0[a]	Total follow-up	Day 0[a]	After Day 0[a]	Total follow-up
Any severe extrarenal involvement	9 (17)	8 (15)	13 (25)	8 (15)	10 (19)	14 (27)
Any severe neurological involvement	6 (12)	4 (8)	8 (15)	6 (12)	0 (0.0)	6 (12)
Seizure	6 (12)	3 (6)	8 (15)	6 (12)	0 (0.0)	6 (12)
Brain infarction	0 (0.0)	1 (2)	1 (2)	0 (0.0)	0 (0.0)	0 (0.0)
Coma	0 (0.0)	0 (0.0)	0 (0.0)	0 (0.0)	0 (0.0)	0 (0.0)
Any severe cardiovascular involvement	1 (2)	3 (6)	4 (8)	2 (4)	5 (10)	7 (14)
Hemodynamic instability	1 (2)	3 (6)	4 (8)	2 (4)	4 (8)	6 (12)
Myocardial failure	0 (0.0)	0 (0.0)	0 (0.0)	0 (0.0)	1 (2)	1 (2)
Myocarditis	0 (0.0)	0 (0.0)	0 (0.0)	0 (0.0)	0 (0.0)	0 (0.0)
Any severe gastrointestinal involvement	4 (8)	1 (2)	5 (10)	2 (4)	2 (4)	4 (8)
Hemorrhagic colitis	3 (6)	1 (2)	4 (8)	2 (4)	1 (2)	3 (6)
Ischemic colitis	1 (2)	0 (0.0)	1 (2)	0 (0.0)	0 (0.0)	0 (0.0)
Pancreatitis	0 (0.0)	0 (0.0)	0 (0.0)	0 (0.0)	1 (2)	1 (2)
Any severe respiratory involvement	2 (4)	5 (10)	7 (14)	1 (2)	6 (12)	7 (14)
Mechanical ventilation	2 (4)	5 (10)	7 (14)	1 (2)	4 (8)	5 (10)
Respiratory distress syndrome	0 (0.0)	2 (4)	2 (4)	0 (0.0)	5 (10)	5 (10)

[a]Day 0 corresponds to the date of the Shiga toxin-producing *Escherichia coli* associated-hemolytic uremic syndrome (STEC-HUS) diagnosis in the participating site

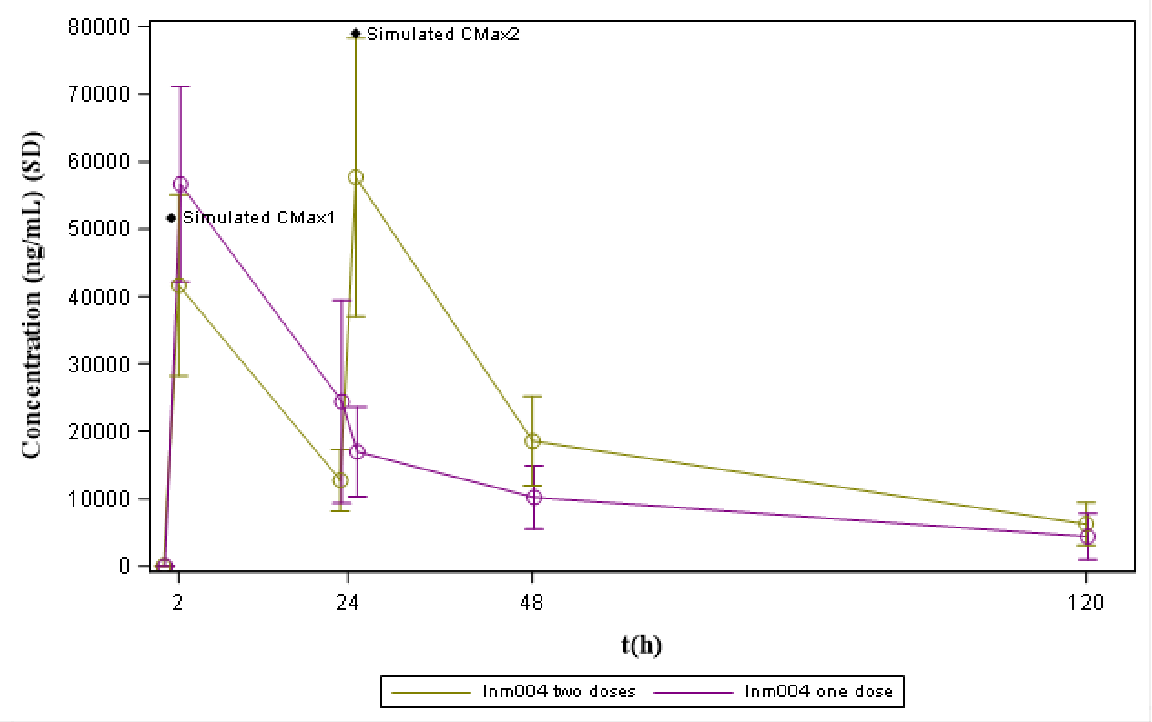

Supplementary Fig. 1. Plasma concentration-time profile of INM004 by dose-regimen.
Cmax1, peak plasma concentration of INM004 after the first dose; *Cmax2*, peak plasma concentration of INM004 after the second dose; *t*, time; *h*, hours
